# Groundwater well optimization to minimize contaminant movement from a surficial shallow aquifer to a lower water supply aquifer using stochastic simulation-optimization modeling techniques: Strategy formulation

**DOI:** 10.1016/j.mex.2022.101765

**Published:** 2022-06-19

**Authors:** Sondipon Paul, Brian Waldron, Farhad Jazaei, Daniel Larsen, Scott Schoefernacker

**Affiliations:** a Department of Civil Engineering, University of Memphis, Memphis, TN 38152, USA; b Center for Applied Earth Sciences and Engineering Research (CAESER), University of Memphis, Memphis, TN 38152, USA; c Department of Earth Sciences, University of Memphis, Memphis, TN 38152, USA

**Keywords:** Simulation-optimization model, Groundwater model, Aquifer interaction, Groundwater contamination, MODFLOW, MODPATH, Genetic algorithm, FloPy, Python

## Abstract

The interaction between surficial shallow aquifers of poorer quality and semi-confined water-supply aquifers poses a potential risk for degradation of the water supply. Groundwater engineers and hydrogeologists use groundwater models to synthesize field data, conceptualize hydrological processes, and improve understanding of the groundwater system to support informed decision-making. Models for decision-making, called management models, aid in the efficient planning and sustainable management of groundwater systems. Management models search for the best or least-cost management strategy satisfying hydrologic and environmental regulations. In management models, a simulation model is linked or coupled with an optimization formulation. Widely used optimization formulations are linear, non-linear, quadratic, dynamic, and global search models. Management models are applied but are not limited to maximizing withdrawals, minimizing drawdown, pumping costs, and saltwater intrusion, and determining the best locations for production wells.

This paper theoretically presents the development of groundwater wellfield management strategies and the corresponding modeling framework for each strategy's evaluation. Depending on the strategy, the modeling effort applies deterministic (simulation) and stochastic (simulation-optimization) techniques. The goals of the optimization strategies are to protect wells from potential contaminant sources, identify optimal future well installation sites, mitigate risks, and extend the life of wells that may face water contamination issues.

• Several management strategies are formulated addressing well depth, seasonal pumping operation, and mapping no-drilling or red zones for new well installation.
• Modeling methodologies are laid down that apply thousands of numerical simulations for each strategy to simulate and evaluate recurring patterns of contaminant movement.
• The simulation model integrates MODFLOW and MODPATH to simulate 3D groundwater flow and advective contaminant movement, respectively and is transferred via FloPy to couple with the optimization/decision model using a custom Python script.

## Graphical abstract

**Figure:**
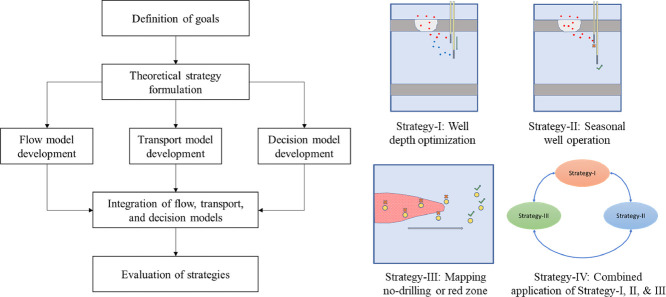

Specifications table

**Table:** 

Subject Area:	Engineering
More specific subject area:	Groundwater flow and contaminant transport, groundwater model, simulation-optimization model, hydrogeology, wellfield management, well optimization.
Method name:	Groundwater production well optimization methods
Name and reference of original method:	(1) Management model development for decision analysis [6]. “Convergence of Stochastic Optimization and Decision Analysis in the Engineering Design of Aquifer Remediation.” Ground Water, 37(6), 934–954. 10.1111/j.1745-6584.1999.tb01193.x, [7]. “Hydrogeological Decision Analysis: 1. A Framework.” *Groundwater*, 28(5), 738–766. 10.1111/J.1745-6584.1990.TB01989.X, (2) Genetic Algorithm based groundwater pumping optimization [17]. “Genetic Algorithm Solution of Groundwater Management Models.” *Water Resources Research*, 30(6), 1897–1906. 10.1029/94WR00554
Resource availability:	N.A.

## Method details

### Overview

Surficial shallow aquifers are vulnerable to anthropogenic contamination due to urbanization, industrialization, and agriculture. Under favorable conditions, the shallow aquifer's contaminated water can migrate into an underlying semi-confined aquifer [5,20]. Therefore, in groundwater systems that comprise a series of unconsolidated aquifers and their aquitards, inter-aquifer exchange between aquifers can influence the water quality [25]. If the semi-confined aquifer is used for water supply, the leakage may contaminate groundwater wells, thereby lowering water quality and resulting in the abandonment of the expensive production wells. The contaminated water migration mechanisms from shallow to a semi-confined aquifer separated by leaky aquitards are relevant to many areas, for example, Toronto, Ontario, Canada [8]; Murray Basin, Australia [27]; and Shelby County, Tennessee USA [14]. The present paper aims to formulate well optimization strategies for managing groundwater wellfields. It also theoretically presents strategy evaluation frameworks using groundwater modeling techniques. The goals of the wellfield management are to solve the well contamination problem posed by hydrogeological breaches or windows [[11], [29]] in the separating confining aquitards lying between the aquifers.

Groundwater models serve as simplified representations of complex natural systems that provide a quantitative framework to synthesize field data, conceptualize hydrological processes and hydrological insights, and provide insight into groundwater response to external stressors. The purposes of groundwater modeling are many, but an intent common to all is an improved understanding of the groundwater system that can serve to support informed decision-making [31]. Management models or models for decision-making aid in the efficient planning and sustainable management of groundwater systems can link or couple a simulation model with an optimization formulation to search for the best or least-cost management strategy subject to hydrologic and environmental regulations [7,^17^,^24^,28]. The simulation model predicts the hydraulic or chemical responses needed to identify the optimal design satisfying the specified constraints in the hydraulic head, flow, gradient, and concentration standards. The optimization algorithm employs intelligent search techniques that traverse the decision space in search for the optimal solution.

### Methods description

A deterministic simulation based management model may suffice to make decision analysis for simpler problems. However, a more complex problem may require simulation-optimization based management models [6]. The simulation-optimization based management models are developed in three different ways [6]: (i) linking flow and transport simulator to the optimization algorithm where the models are solved sequentially without feedback within the time step; (ii) coupling flow and transport simulator to the optimization algorithm where the models are solved iteratively within the time step, and input to each model is updated to reflect output from the other, and (iii) embedding flow and transport simulator to serve as constraints to the optimization algorithm. This paper's theoretical modeling methodology applies deterministic (simulation) and stochastic (simulation-optimization) techniques, depending on the strategy.

A variety of optimization formulations and corresponding solution methods are available. Widely used optimization formulations are linear, quadratic, dynamic, stochastic, non-linear models, and global search algorithms [1,30]. The Genetic Algorithm [9] is a stochastic global search procedure based on the theory of evolution called natural selection, hypothesized by Charles Darwin. It applies stochastic transition rules to generate a population by selecting the fittest individuals from the previous population. In this paper, the genetic algorithm is deployed as the optimization program due to its efficiency in finding global or near-global optimum solutions. The formulation of the genetic algorithm is straightforward, and simpler models can employ quick solutions (i.e., linear). However, more complicated problems (e.g., discontinuous or highly non-linear and nonconvex problems) can be computationally expensive [10,30]. In the latter situation, solving the genetic algorithm on a parallel system lowers the simulation time required to complete each iteration [17]. Since evaluating each chromosome's fitness in genetic algorithm is inherently independent, each evaluation process is simulated simultaneously by distributing it to separate parallel processors. The genetic algorithm is used in groundwater literature to solve problems like well location and pumping rate optimization [12,^13^,26], aquifer remediation [10,^16^,^17^,23], and parameter estimation [15].

Khalaf and Gad [12] use Visual MODFLOW as the simulation model and a genetic algorithm constructed with Fortran as the optimization program. Huang and Mayer [10] develop a simulation-optimization model to find the optimal solution by coupling a hypothetical MODFLOW model with the genetic algorithm optimization process. Maskey et al. [16] couple MODFLOW and MODPATH with a genetic algorithm. Madsen and Perry [15] code a genetic algorithm using Visual Basic script and apply it to a MODFLOW model. The groundwater modeling frameworks of this paper use MODFLOW-NWT [19] as a flow simulator. MODPATH 7 [22] is used to simulate the advective transport of modern water (age <60 years) as the age of water is not affected by retarding physical, chemical, and biological processes. FloPy, a Python package for developing, running, and postprocessing MODFLOW models [3,4], is used to automate the construction of MODFLOW and MODPATH models based on various inputs, both user-defined and generated based on progressive genetic algorithm results. Since an automation process is needed that would run MODFLOW and MODPATH recursively and couple the flow and transport simulator as an independent function to the optimization code, FloPy is utilized to build the models. The Python multiprocessing library is utilized to run all processes in an iteration concurrently.

The primary assumption of the presented well optimization problem is that the potentially contaminated modern water is migrating from the surficial shallow aquifer to an underlying semi-confined aquifer used for water supply through breaches in the intervening aquitard. The goals of the well optimization are to protect production wells from contamination, identify suitable locations for future wells, and extend the life of wells. To achieve the goals, four management strategies are formulated. The formulated strategies are (1) Strategy-I: well depth optimization; (2) Strategy-II: seasonal pumping operation; (3) Strategy-III: mapping no-drilling or red zone for new well installation; and (4) Strategy-IV: combined application of Strategy-I, II, and III or synchronized optimization. Detailed discussions on the formulated strategies are presented in the following sections. A projected simulation period is required to choose to ensure protection for the production wells over the design life. A typical public water supply well's lifespan is about 40 to 50 years, and at the end of this period, abandonment or reinstalment is performed [18]. It is also likely in certain circumstances that physical, biological, or chemical processes may neutralize contaminants during this time period [21]. Therefore, a projected simulation period of 50 years can be selected for model runs and evaluation purposes.

#### Strategy-I: well depth optimization

**Fig. 1:**
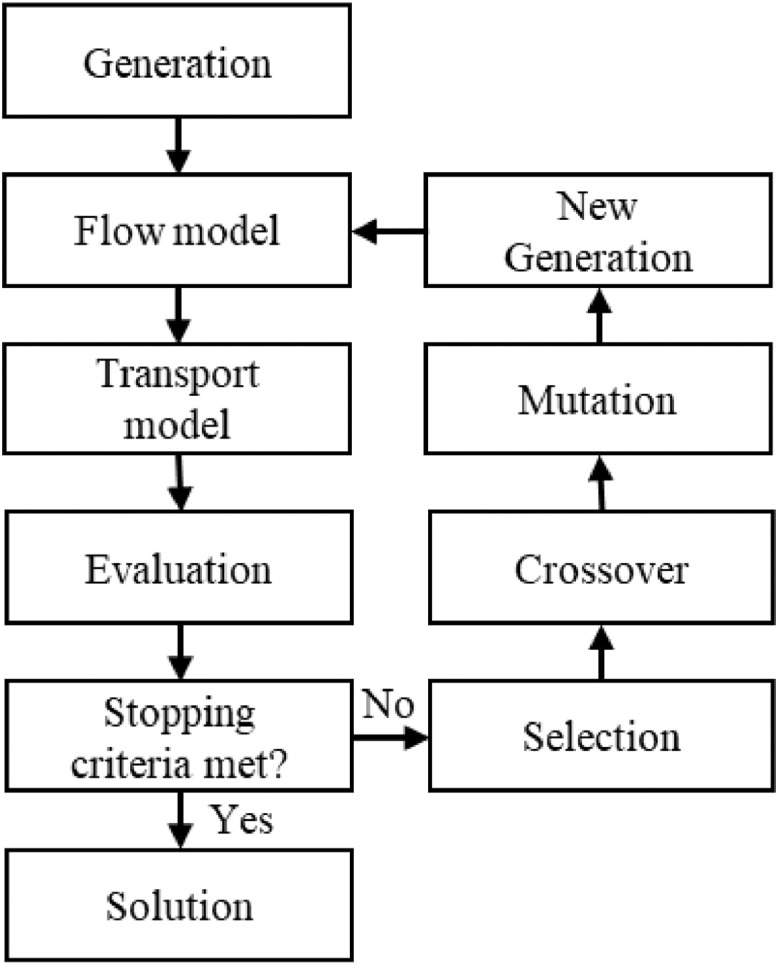
Major steps in Strategy-I: Well depth optimization.

Well depth optimization strategy is applicable when the water supply aquifer is thick enough to compartmentalize into two or more sections. In the strategy, production wells screened in the upper section of the semi-confined aquifer are moved to lower sections of the aquifer. The relocation may reduce the movement of modern water to the upper semi-confined aquifer or determine if increasing overall withdrawal from deeper aquifer sections result in modern water at these lower depths. Hence, the employment of the simulation-optimization technique (Fig. 1) is used for the strategy. MODFLOW simulates flow, and MODPATH simulates advective contaminant transport using forward particle tracking method. Particles begin in the breach within the separating confining layer lying between shallow and semi-confined aquifer. The ‘stop_at’ option for weak sinks/sources is used in MODPATH as a particle entering a cell with a well serves as the trigger for modern water reaching the screen.

A finer vertical discretization of the water supply aquifer is needed in the flow model for the depth optimization strategy because the objective of Strategy-I mandates placing well screens at various depths. When a model layer is divided into finer vertical discretization, well screens may span over multiple numerical layers. Instead of distributing a well's withdrawal according to the portion of screen length within a layer, total withdrawal is lumped into the uppermost cell of the spanned layers except when 70% (arbitrary) of the extraction occurred in the lower layer to which the total withdrawal is lumped. Placing total withdrawal within a single layer helps simplify the optimization (e.g.,., adopting layer numbers as the decision variables) process and by prioritizing the upper layer to remain conservative in the test for potential modern water contamination at the well.

In the optimization process, well screen depths are optimized within a wellfield where the number of particles from a nearby breach (or breaches) is minimal or absent. The objective function is defined as the summed particle count at the well screens of the wellfield under investigation. Genes represent the model layer (top numerical layer to bottom numerical layer modeled as the semi-confined aquifer) and act as the decision variables in the genetic algorithm process. The genetic algorithm generates multiple well screen realizations, and FloPy enacts MODFLOW and MODPATH runs for each realization. Within each iteration, the optimization algorithm examines model results to identify a set of improved solutions and passes the better solutions to the subsequent generation until optimization criteria are met.

The genetic algorithm optimization process for the well depth optimization strategy is defined as:

**Table:** 

Objective Function:	Minimize ∑ Number of particles terminating at each well of the studied wellfield
Decision Variable:	Layer number associated with the well screen depth
Constraint:	Maximum Layer Number = Bottom layer of the semi-confined aquifer Minimum Layer Number = Upper layer of the semi-confined aquifer)

Two stopping criteria are used: (i) a maximum number of allowable iterations, or (ii) the objective function = 0. When a solution (well-depth stratification regime) returns zero particles, the process stops, and the regime is taken as the optimized solution. Otherwise, the simulation continues up to the maximum allowable generation, and the solution returning the lowest summed particle count is taken as the optimized solution. The number of maximum iterations depends on the complexity of the problem and can be approximated by experience. The Python multiprocessing package for the parallel simulation is used to distribute parallel optimization runs on a computer cluster to reduce computation time.

#### Strategy-II: seasonal well operation

As expected for municipal consumption, water demand fluctuates by season, where more water resources are extracted in warmer periods. This seasonal pattern can be used to potentially reduce the movement of modern water during seasons of low water demand (i.e., winter and spring) by turning off wells screened in the upper section of the semi-confined aquifer and withdrawing groundwater from deeper section wells in the aquifer. The strategy assumes that the deeper wells can run at full capacity to meet the water supply demand of the wellfield. In this strategy, upper semi-confined aquifer wells are wells that pump 30% or more (arbitrary) from upper one-third of the aquifer's total depth. In contrast, the wells pumping 70% or more (arbitrary) from lower two-thirds of the aquifer are lower semi-confined aquifer wells. The low-demand period can be determined by comparing each monthly stress period's pumping rate with the 25th percentile pumping rate. The 25th percentile pumping rate is calculated from the stress period's pumping rate data of the studied wellfield.

**Fig. 2:**
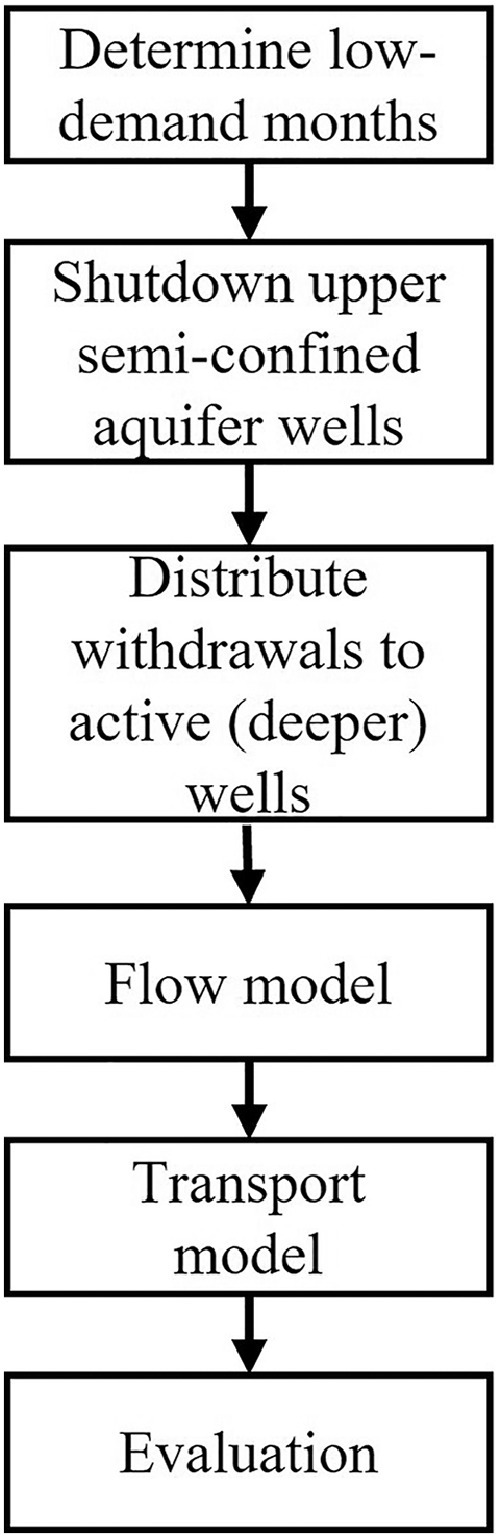
Major workflow in Strategy-II: Seasonal well operation.

Withdrawals from the deactivated upper semi-confined aquifer wells are redistributed among the active semi-confined aquifer wells screened within the deeper section of the aquifer. Like before, particles are placed within cells representing breaches in the confining aquitard, and well-screen bearing cells (sink/source cells) in the semi-confined aquifer are defined as the particle termination locations. The ‘stop_at’ option for weak sinks/sources is defined for the particle tracking simulation. The strategy estimates the number of particles received by the wells and generates particle path lines for evaluation after the projected simulation period of 50 years. The major steps in Strategy-II are shown in Fig. 2.

#### Strategy-III: mapping no-drilling or red zone

The goal of the location optimization strategy is to identify locations of minimal modern water contamination for future groundwater production wells installation. The risk of modern water contamination via the separating confining layer is minimized or non-existent at these identified locations. The safe new well locations are delineated through examination within a predefined zone. The predefined zone is delineated by encompassing the studied wellfield, its nearby breaches, and the distance that particle may travel over the projected simulation period (i.e., particles remain within the zone). In this strategy, a multilayer well regime can be used. In multilayer well regimes, well screens are spanned over multiple numerical layers.

**Fig. 3:**
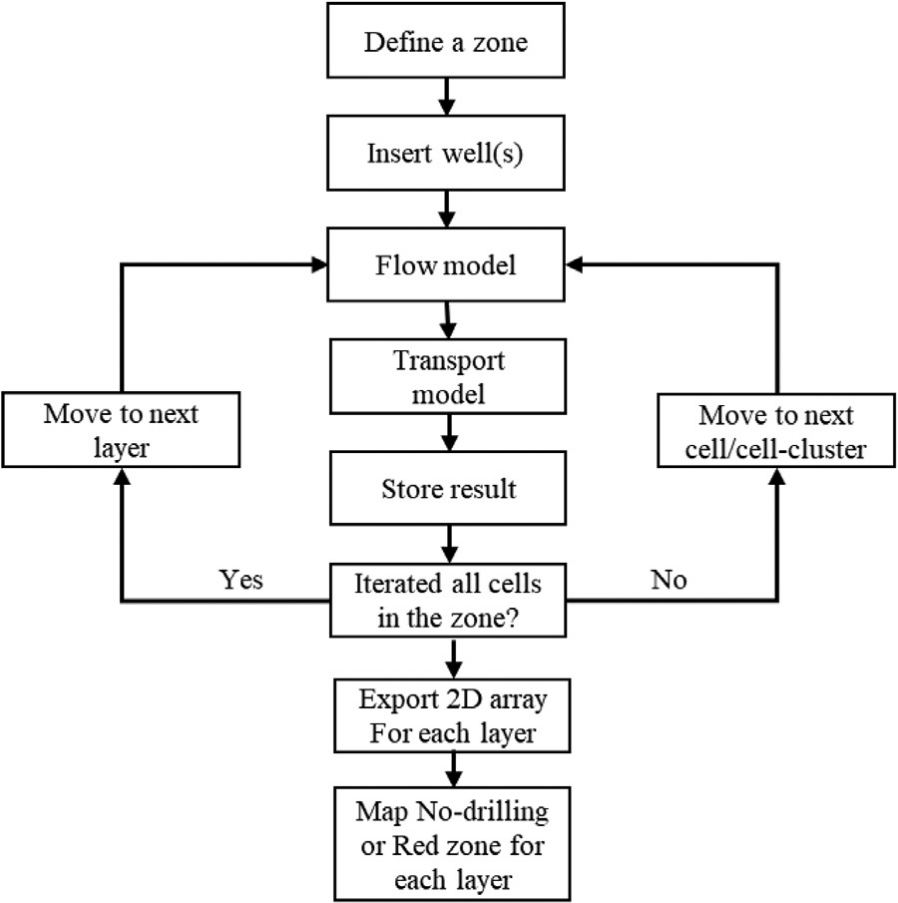
Major workflow in Strategy-III: mapping no-drilling or red zone.

Using FloPy, the scripted deterministic MODFLOW-MODPATH simulation code traverses a new well in the predefined zone, systematically placing the well in each unoccupied cell (i.e., no pre-existing well). A constant pumping rate is applied to the new well where the assigned rate can be taken from the highest extraction rate of the stress period data for the wellfield under consideration. Like the depth optimization strategy, particles are generated at the center of the separating confining layer breach cells. The cell hosting the moving well is defined as the particle termination location in this strategy. After the simulation, the number of particles that terminates at the moving well cell is determined. Fig. 3 depicts the workflow of Strategy-III.

The analysis includes screening the new well within each layer (from the top to the bottom layer of the semi-confined aquifer) before moving the well to the next unoccupied cell. The predefined zone is defined to reduce the total number of traversing cells to limit the overall simulation time for the strategy. Yet, the iteration of the well in the zone for the optimization process may need thousands of model runs per layer depending on the number of model grids covered by the zone. Only the installation of a single well is tested in the strategy, not the insertion of two or more wells at a time. Exported model results are gridded using a scripted Geographical Information System postprocessing tool within ESRI® ArcGIS Pro, and contour maps are generated. These maps delineate the extent of leakage migration within each layer simulated as the semi-confined water supply aquifer for the studied wellfield outside of which construction of future wells can be assumed safe over the projected future.

#### Strategy-IV: combining Strategies-I, II, and III or synchronized optimization

**Fig. 4:**
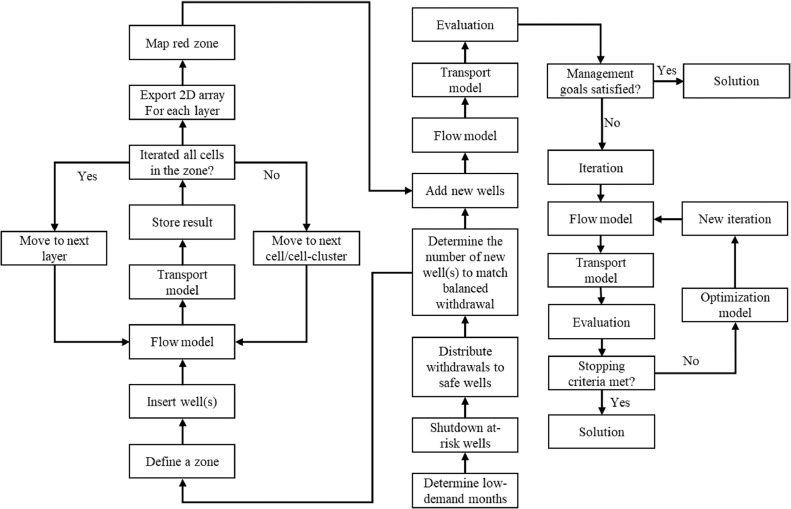
Workflow of Strategy-IV: synchronized optimization.

The synchronized optimization analysis simultaneously applies location, seasonal, and depth optimization strategies to provide an improved solution for the wellfield management problem. The workflow for Strategy-IV is shown in Fig. 4. All upper semi-confined aquifer wells are turned off in Strategy-II irrespective of the well's distance from breaches. In contrast, upper and lower semi-confined aquifer wells are turned off if they are proximal (at-risk) to the breach during the low water demand period for Strategy-IV. The withdrawals of the inactive wells are redistributed among the remaining active wells to maintain water production at the wellfield.

Based on the well capacity, additional production wells may be required to maintain water production of the studied wellfield, followed by turning off the endangered wells during the low demand months. The well location optimization (Strategy-III) modeling framework is implemented at this stage to map safe locations for the new wells to place. For simplicity, the additional wells are screened within a single model layer.

During the high-demand period of the year, the new wells also remain active along with the pre-existing wells. The total wellfield withdrawal is equally distributed over all the active wells (new and pre-existing). The distribution of pumping among the new wells decreases the pumping load from the pre-existing wells during the high demand period. The strategy uses the deterministic MODFLOW-MODPATH based management modeling framework to evaluate the effect of seasonal well operation on modern water contamination, followed by the stochastic depth optimization simulations based on MODFLOW, MODPATH, and genetic algorithm modeling framework.

## Method validation

### Conceptual model

A conceptual problem is solved to validate the simulation-optimization computer code developed for the well depth optimization strategy, a process common in new code development [10,^13^,^16^,^17^,^23^,26]. A simplified model is tested to reduce the number of optimization parameters and reduce the time to convergence. The conceptual model is taken from a problem (P.4.3) posed in Anderson et al. [2] of a simple two-dimensional, valley-fill aquifer of homogeneous material, simple model boundary conditions, and steady-state flow. The valley has its northern and southern boundaries defined as a river and a swamp, respectively, with impervious mountains along the east and west boundaries.

### Numerical model

**Fig. 5:**
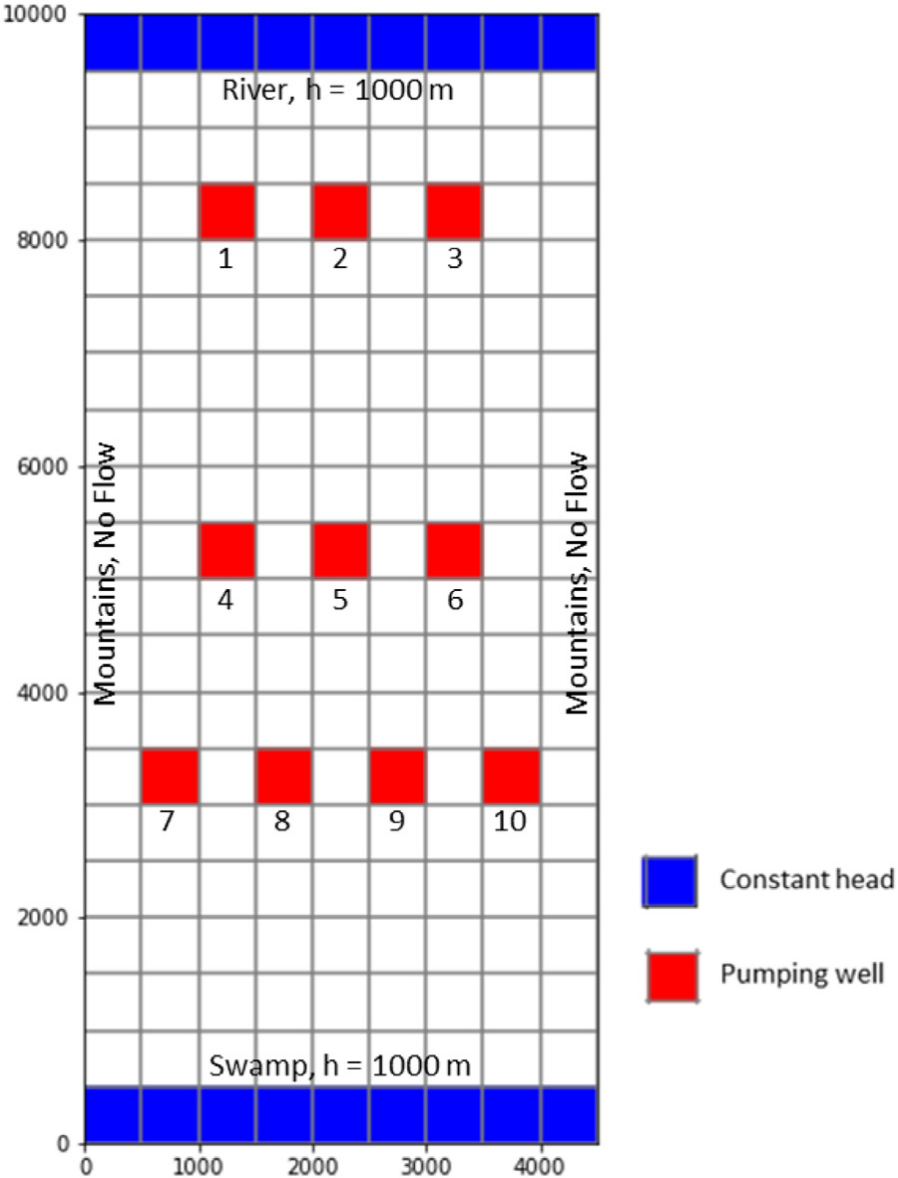
The model domain map shows the finite-difference grid, pumping wells, and boundary conditions for the pumping rate optimization example (modified from Anderson, Woessner, and Hunt [2]).

The pumping regime concept is adopted from McKinney and Lin [17] for insertion into the conceptual model. The model is constructed using FloPy, as it is used to develop enumerable well configurations in Strategy-I. The numerical model (Fig. 5) has an east-west dimension of 4.5 km and a north-south dimension of 10 km. Square grids of 500 m discretize the model domain into 20 rows and 9 columns. A uniform unconfined layer thickness of 50 m is assumed throughout the model. Recharge is a uniform 0.001 m/d, hydraulic conductivity is 50 m/d, and the aquifer is homogenous/isotropic. The river and the swamp are represented by a constant head boundary of 1000 m, while the east and west are specified no-flow boundaries. A steady-state, two-dimensional flow condition is considered assuming that the pumping wells would be operated for an infinite period.

### Optimization model

The linear management problem from McKinney and Lin [17] is utilized to test the model code. The objective of the problem formulation is to maximize pumping rates at ten active wells operating in the unconfined aquifer. The genetic optimization algorithm code is modified, assigning pumping rates as the decision variable, contrasting the well depth optimization problem where well screen layers (surrogate to well screen depths) are assigned as decision variables. Additionally, the present optimization problem defines the summation of pumping rates at the wells as the objective function, unlike the well-depth optimization problem where total received particles are defined as the objective function. Here, the management model maximizes groundwater production from the aquifer by satisfying two constraints: the pumping rate cannot exceed 7000 m^3^/d, and cells containing wells cannot go dry. The genetic algorithm optimization process comprises a population of 100 members (chromosomes) where each member contains ten genes representing pumping rates at the ten wells. Elitism method is applied to select parents, a one-point crossover method at a random location is implemented to generate offspring, and a mutation rate of 0.01 (=1/Population [17] is adopted to ensure diversity in the genetic algorithm process. The mathematical expression of the pumping rate optimization problem is given below:

**Table:** 

Objective Function:	Maximize $\sum_{i=1}^{10}Q_{i}$
Decision Variable:	Pumping rates, Q_i_; *i* = 1 to 10
Constraint:	0 ≤ Q_i_ ≤ 7000 m^3^/d; *i =* 1 to 10 h_i_ ≥ 980 m
Stopping Criteria:	100 iterations

## Results

**Fig. 6:**
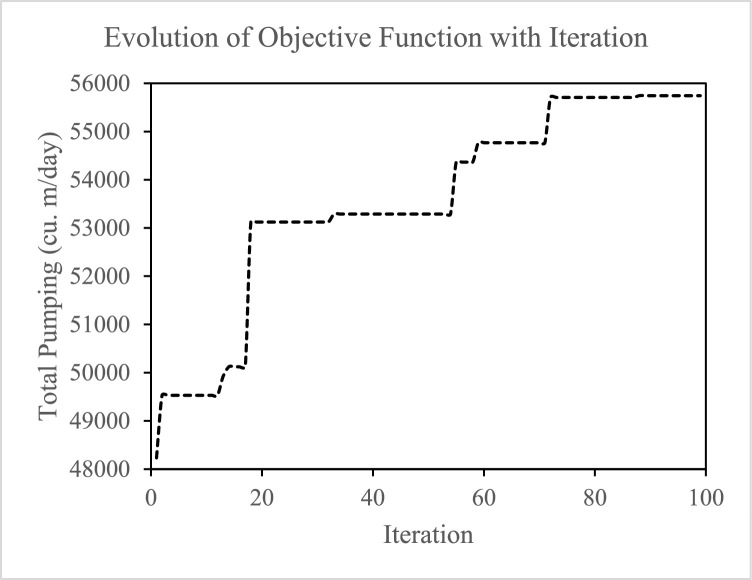
Changes in the objective function with elapsed iteration.

The convergence of the objective function with elapsed iteration during the simulation-optimization process is shown in Fig. 6. The solution continues to improve until near the 75th iteration, when it becomes stable, showing no significant improvement. The results of the present simulation-optimization process are compared with the results in McKinney and Lin [17] (Table 1), and the analogy indicates that the summed optimized pumping rates obtained using the present model is four percent less than the cumulative withdrawal of the genetic algorithm process of McKinney and Lin [17]. For the southern well cluster (wells 7-10), the distribution of pumping in the result is notably different than McKinney and Lin [17] and are not symmetric as indicated in the simplex and genetic algorithm methods of McKinney and Lin [17].

**Table 1:** A comparison of pumping rate maximization results with the results from McKinney and Lin [17].

Well	Results from McKinney and Lin [17]		Present work
	Rate from simplex method (m3/d)	Rate From Genetic Algorithm (m3/d)	Rate From Genetic Algorithm (m3/d)
1	7000	7000	6298
2	7000	7000	6579
3	7000	7000	5715
4	6000	7000	5351
5	4500	2000	4775
6	6000	6000	5920
7	6800	7000	3633
8	4100	4000	6092
9	4100	4000	6338
10	6800	7000	5042
Total	59,300	58,000	55,743

In the genetic algorithm code in McKinney and Lin [17], the decision variables are coded as three-digit binary substrings (minimum value: binary 000 = decimal 0; maximum value: binary 111 = decimal 7). Therefore, the decision variables can accommodate only integer values ranging from 0 to 7 with an increment of 1. This implies that the pumping rates at the wells can only be 0, 1000, 2000,…,7000 m3/d at an increment of 1000 m3/d, contrasting the developed genetic algorithm code of this paper takes decimal values (i.e., continuous decision variables instead of binary) which accommodates alteration at its 10th place. Additionally, the code employs tournament selection by elitism method and a one-point crossover process at random locations of the chromosome. The difference in the code structure likely explains the variation in the solution of the optimization process. When using a genetic algorithm, the reproducibility of results is not guaranteed due to its random search process. The search returns a near-global solution that is expected to vary from process to process, which explains the asymmetry about wells 7-10 in the result. Also, the selection of the iteration number depends on the complexity of the problem and the desired accuracy at the expense of computational effort.

The goal of the conceptual optimization problem is to maximize groundwater production from the conceptual aquifer. Therefore, cumulative withdrawal at the ten wells defines the objective function in the optimization formulation. The optimization process evaluates a population of 100 chromosomes having ten genes in each chromosome per generation. The elitism method is applied to select fitter parents for crossover and assign a mutation rate of 0.01 to ensure diversity in the search process. No cell drying at the wells and a maximum allowable pumping rate of 7000 m3/d are the two management criteria that the process satisfies. McKinney and Lin [17] suggest that a finer discretization of the decision variables with additional substrings (members in a population) can provide a more accurate solution. McKinney and Lin [17] apply 1000 m3/d discretization to the decision variable compared to the present process that accommodates changes at the 10th place. Moreover, 100 chromosomes are employed instead of 64 chromosomes of McKinney and Lin [17]. The optimization process stabilizes in 10 generations for McKinney and Lin [17] because of the large discretization. In contrast, the developed process of this paper needs more iterations (75 iterations) to stabilize.

**Fig. 7:**
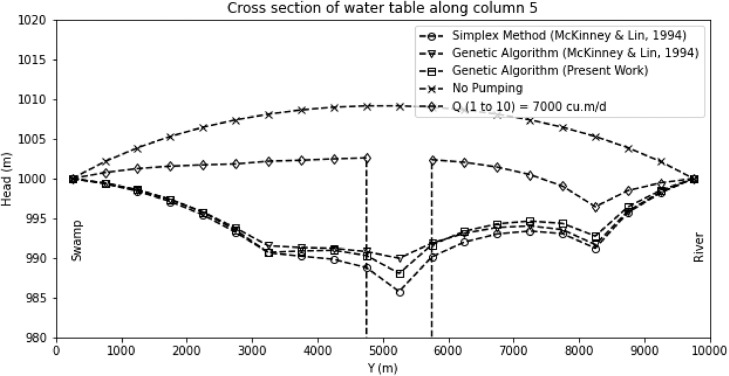
Head distribution along column five for different pumping regimes, including the base condition (no pumping) and all wells running at capacity (10 × 7000 = 70,000 m^3^/day).

A finer discretization of the decision variables with a larger population size suggests that the presented optimization model offers an improvement over the simulation-optimization model of McKinney and Lin [17] with a more robust result. The head cross-section along the model's fifth column is compared to visualize the effect of different pumping conditions (Fig. 7). Note that when all the pumps are extracting at their maximum allowable rate (i.e., 7000 m^3^/day), cell drying occurs. The head distribution for the pumping rates obtained from different optimization processes are clustered along with a narrow band; therefore, it is concluded that the developed simulation-optimization code can produce a near-global solution for the groundwater pumping maximization problem. It may also be inferred that the algorithm originally developed to solve the well depth optimization problem would also produce the best solution for the problem.

## Summary and discussion

The four management strategies and associated modeling frameworks can provide practical solutions to the well contamination problem due to modern water inflowing from a surficial shallow aquifer to a semi-confined aquifer. The utility of the developed four strategies for wellfield management are summarized below:

### Strategy-I

The well depth optimization strategy relocates well screens vertically downward to exploit the safe lower section of a semi-confined aquifer of sizable thickness. The strategy also considers minimizing the implementation cost by reducing affected wells. The objective of the strategy is to protect pre-existing wells from contamination for a projected period in the future, typically the design life of the groundwater wells. The strategy is useful for wellfields as the current lands can be used for relocating the pre-existing wells to a deeper section of the water supply aquifer. Therefore, costs associated with land acquisition and threats from surrounding unexplored contamination sources (i.e., breach) are minimized.

### Strategy-II

The seasonal well operation strategy schedules turning off upper semi-confined aquifer wells as modern water from the contaminated shallow aquifer remains within the upper production zone of the semi-confined aquifer. The strategy exploits the seasonal variation of water demand since the winter and spring months consume less water than the summer months. The strategy intends to relax modern water encroachment and extend the life of potentially at-risk (i.e., upper semi-confined aquifer wells) pre-existing wells. The strategy does not warrant an additional cost because no drilling and land acquisition is needed for its implementation.

### Strategy-III

A water supply utility may need to install a new well or multiple wells due to several reasons- maintaining increased demand, contamination, and end of life of the pre-existing wells. The strategy focuses on protecting against contamination of the new wells. The no-drilling or red zones for each wellfield surrounding its contamination sources (breaches) theoretically delineate the minimal contamination impacted areas in the horizontal plane for each numerical layer (i.e., at various depths) simulated as the semi-confined water supply aquifer. The red zone maps would help the concerned utility to select appropriate locations and depths for new well installation, evaluating other facts such as land use, city planning, contamination sources, and hydraulic properties.

### Strategy-IV

Strategy-I and II depend on wellfield-specific characteristics such as a wells' configuration (e.g., the ratio of deep and shallow wells) and their distances (distribution densities) around known or potential breaches. In contrast, Strategy-III does not consider the effect of altered flow dynamics (due to adding new wells in a wellfield) on the contamination potential to the pre-existing wells. Strategy-IV is more conservative as it assumes a lower pump capacity and considers wells located in proximity to a breach as vulnerable. Although Strategy-IV offers a better solution as it addresses and eliminates the shortcomings of the individual approaches, it could be more expensive to implement as drilling, land acquisition, and other infrastructure developments (i.e., water supply mains to treatment plants) are required.

The selection of a flow model depends on its spatial and vertical resolution, hydrogeologic and hydraulic features, and ability to generate desired output in the context of the management models. A finer horizontal and vertical resolution of the flow model is desired to evaluate Strategy-I and to improve the accuracy of transport code simulations. In contrast, the selection of transport modeling code depends on the contaminant transport processes in the studied aquifer system. Advective transport dominates modern water migration to a semi-confined aquifer than dispersion, reaction, and retardation processes. Therefore, particle tracking simulations are adopted to evaluate useful information for decision analysis in this paper.

The integration of optimization models with the flow and transport models depends on cases when a spectrum of solutions is required to satisfy certain physical and technical measures from which the best solution needs to be identified. The general task of an optimization model is to identify the best solution by minimizing/maximizing a mathematical expression called the objective function formed by decision variables. Besides an objective function(s), it is critical that some constraints be defined based on which the optimization model would optimize the objective function. Strategy-II does not require meeting any constraints based on physical or technical regulations because only pumping rates are being shifted from shallow wells to deeper wells. Regarding Strategy-III, it only requires land access which is not constrained. Therefore, a flow model linked to a transport simulator can evaluate Strategy-II and III. In contrast, Strategy-I and IV require a simulation-optimization model because lowering a well screen can result in contaminating other wells, whether shallow or deep; hence, optimization is needed to choose the best configuration from the several possible well configurations.

The modeling framework is developed utilizing the powerful programming platform Python and FloPy. FloPy is a Python library that allows simulating MODFLOW and its related codes in the Python environment. Besides building flow and transport models using FloPy, groundwater modelers can use Graphical User Interface (GUI) based modeling software to build flow models and load into FloPy to run the models programmatically. The application of Python and FloPy in model development is particularly useful because decision tools (i.e., an optimization model or a custom simulation-based decision tool) can be conveniently developed using Python codes and integrated with the FloPy-based simulator models. The platform also offers great efficiency since multiple processes can be performed parallelly using the Python multiprocessing library, reducing simulation time significantly. The platform also presents great flexibility to the modeling framework since it can be modified easily to solve any groundwater management problem.

## Data availability statement

Some or all data, models, and results that support this study are available from the corresponding authors and the Center for Applied Earth Science and Engineering Research (CAESER) upon reasonable request.

## Declaration of Competing Interest

The authors declare that they have no known competing financial interests or personal relationships that could have appeared to influence the work reported in this paper.

## Data Availability

Data will be made available on request.
